# Quantitative Assessment of Blood Lactate in Shock: Measure of Hypoxia or Beneficial Energy Source

**DOI:** 10.1155/2020/2608318

**Published:** 2020-10-14

**Authors:** David G. Levitt, Joseph E. Levitt, Michael D. Levitt

**Affiliations:** ^1^Department of Integrative Biology and Physiology, University of Minnesota, 6-125 Jackson Hall, 321 Church St. S. E., Minneapolis, MN 55455, USA; ^2^Division of Pulmonary and Critical Care Medicine, Stanford University, 300 Pasteur Drive, Stanford, CA 94305, USA; ^3^Research Service, Veterans Affairs Medical Center, VAMC/111D, 1 Veterans Drive, Minneapolis, MN 55417, USA

## Abstract

Blood lactate concentration predicts mortality in critically ill patients and is clinically used in the diagnosis, grading of severity, and monitoring response to therapy of septic shock. This paper summarizes available quantitative data to provide the first comprehensive description and critique of the accepted concepts of the physiology of lactate in health and shock, with particular emphasis on the controversy of whether lactate release is simply a manifestation of tissue hypoxia versus a purposeful transfer (“shuttle”) of lactate between tissues. Basic issues discussed include (1) effect of nonproductive lactate-pyruvate exchange that artifactually enhances flux measurements obtained with labeled lactate, (2) heterogeneous tissue oxygen partial pressure (Krogh model) and potential for unrecognized hypoxia that exists in all tissues, and (3) pathophysiology that distinguishes septic from other forms of shock. Our analysis suggests that due to exchange artifacts, the turnover rate of lactate and the lactate clearance are only about 60% of the values of 1.05 mmol/min/70 kg and 1.5 L/min/70 kg, respectively, determined from the standard tracer kinetics. Lactate turnover reflects lactate release primarily from muscle, gut, adipose, and erythrocytes and uptake by the liver and kidney, primarily for the purpose of energy production (TCA cycle) while the remainder is used for gluconeogenesis (Cori cycle). The well-studied physiology of exercise-induced hyperlactatemia demonstrates massive release from the contracting muscle accompanied by an increased lactate clearance that may occur in recovering nonexercising muscle as well as the liver. The very limited data on lactate kinetics in shock patients suggests that hyperlactatemia reflects both decreased clearance and increased production, possibly primarily in the gut. Our analysis of available data in health and shock suggests that the conventional concept of tissue hypoxia can account for most blood lactate findings and there is no need to implicate a purposeful production of lactate for export to other organs.

## 1. Introduction

Important clinical decisions are based on the observation that high blood lactate levels strongly correlate with mortality in patients with shock from any cause [1], including sepsis [2–4], trauma [5], and myocardial infarction [6]. Despite the immense literature on this topic, including many reviews [7–14], the pathophysiology underlying the correlation between lactate and mortality remains controversial. In large part, this controversy reflects debate concerning the more fundamental question of what is the metabolic role of lactate in health and disease—is lactate simply a by-product of anaerobic metabolism or does it subserve a variety of important physiological functions, in particular as a shuttle delivering an energy source between organs? The present review provides what we believe to be the first in-depth critical evaluation of the experimental evidence supporting each of the two competing concepts of the role of lactate in mammalian physiology. This information is then utilized in an attempt to unravel the complex pathophysiology linking mortality and blood lactate in shock.

In this review, “lactate” represents the L(+)-lactate anion, the predominant enantiomer produced in mammalian metabolism (for a recent comprehensive review of the D(-)-lactate stereoisomer pathophysiology, see [15]). At physiological pH (7.4), more than 99.9% of lactate (pKa = 3.86) is in the ionized form. Although lactic acid is a strong acid, acidosis and blood lactate only weakly correlate [4], and the details of this relationship are controversial. For example, it has been asserted that glycolysis results in a net production of just the lactate anion, not the acid [8, 9]. In any case, we will primarily focus on the biochemistry of the anion and not directly discuss the acidosis. Although blood lactate varies physiologically, increasing after a high carbohydrate meal by as much as 2.5-fold [16], what is usually reported is the basal postprandial value for which we will assume a normal value of 0.7 mM.

The basic metabolic pathways coupled to lactate are simple. Glycolysis of glucose to pyruvate with the net production of 2 ATP occurs in the cytosol:

(1) $$\begin{array}{c} \text{D}‐\text{glucose}+2\text{ NA}{\text{D}}^{+}+2\text{ ADP}+2\text{ Pi}\to \to 2\text{ pyruvat}{\text{e}}^{-}+2\text{ NADH}+2\text{ ATP}+2\;{\text{H}}^{+}+2\;{\text{H}}_{2}\text{O} \end{array}$$

Also, in the cytosol, pyruvate can be reversibly converted to lactate by lactate dehydrogenase (LDH) with the NADH recycled back to NAD^+^:

(2) $$\begin{array}{c} {\text{Pyruvate}}^{-}+\text{NADH}+{\text{H}}^{+}\;\overset{\text{LDH}}{⇄}\text{ lactat}{\text{e}}^{-}+\text{NA}{\text{D}}^{+} \end{array}$$

In anaerobic conditions, the coupling of these two reactions results in the metabolism of glucose to two lactates with the net production of 2 ATP. In the presence of sufficient oxygen, pyruvate is transported into the mitochondria where it is oxidized to CO_2_ and water with the production of 17 ATP per pyruvate (TCA cycle).

Historically, lactate had been regarded as a hypoxic “waste” product released from anoxic tissues that is then either recycled back to glucose in the liver (Cori cycle) or oxidized (by conversion to pyruvate and entering the TCA cycle in mitochondria) in the liver and other tissues [9]. We will refer to this classic view as the “hypoxic” model. In the last 30 years, there has been a major shift in this paradigm resulting from measurements of blood lactate turnover using tracer-labeled lactate. These measurements seemingly show that lactate has a surprisingly rapid turnover under basal (as well as hypoxic) conditions with a rate per mole that is 2.5 times that of glucose in the mouse [17] and 1.5 times in the human (Table 1) and that tissues such as skeletal muscle are simultaneously consuming and producing lactate. It also has been argued that only a small fraction of lactate turnover is associated with the metabolism of hypoxic tissue. Instead, it is proposed that, in the absence of hypoxia, glycolysis can be “directed” to produce lactate for export to other tissues where it serves multiple functions, e.g., as a means by which cells exchange a carbohydrate-derived energy source (the “lactate shuttle”) and as a modulator of important biological functions such as metabolic signaling, inflammation, transcription factors, and angiogenesis [8]. It follows that lactate and pyruvate are primary end products of glycolysis [18, 19]. Evidence for this view is that lactate turnover increases dramatically during exercise with large net lactate flux between resting and exercising muscles (discussed in detail in Exercise). We will refer to this viewpoint as the “nutritive” model.

Although this “nutritive” model is clearly favored in the recent lactate literature, the “hypoxic” model still dominates much of the discussion of the relationship between lactate and shock. For example, a report published in 2015 by the Surviving Sepsis Campaign group [2] makes the inherent assumption that the blood lactate provides a quantitative marker of the level of tissue hypoxia. In contrast, it has been suggested by the proponents of the nutritive model that hypoxia is of minor importance, or even nonexistent, in septic shock and that the increased blood lactate is providing a beneficial metabolic energy source [9–11]. We believe it is important to carefully discuss the experimental basis and clinical implications of these two models since elucidating the shock-lactate correlation requires appropriate modeling of the physiological role of lactate. An example of the confusion on this topic is a recent publication concerning septic shock which states in the Introduction that “… lactate elevation arises from tissue hypoxia” and then, in the Discussion, “… that the controversial lactate shuttle theory … fits well with the bulk of our findings,” both of which cannot be true [4].

Several important issues with regard to the modeling of lactate physiology have received limited discussion. Firstly, the unidirectional tracer lactate fluxes, which are at the heart of the “nutritive” model, may not represent distinct metabolic processes but, rather, nonmetabolic, rapid lactate-pyruvate exchange (Eq. (2)). This is discussed in detail in the Lactate-Pyruvate Exchange. Secondly, a major tenet of the nutritive model is that tissue partial oxygen pressure (pO_2_) measurements have not demonstrated the degree of hypoxia (less than 1 mm Hg pO_2_) required to limit pyruvate oxidation. However, the well-established heterogeneity of tissue pO_2_ could produce localized hypoxic regions that would not be detected by the referenced tissue pO_2_ measurements (discussed in The Krogh Model and Heterogeneity of Tissue O_2_ Partial Pressure). Thirdly, although proponents of the nutritive model have argued that the increased lactate metabolism in exercise might be representative of that in septic shock (i.e., a “hypermetabolic state”), there are marked differences between the exercise and shock states (discussed in Septic Shock and Hemorrhagic and Cardiogenic Shock). Surprisingly, for such an important and well-studied field as shock and lactate, the experimental physiological data on this topic is limited and insufficient to definitively answer many important questions. In this paper, we have attempted to summarize and evaluate all relevant information on the pathophysiology of lactate in shock.

## 2. Lactate Biochemistry

**Figure 1:**
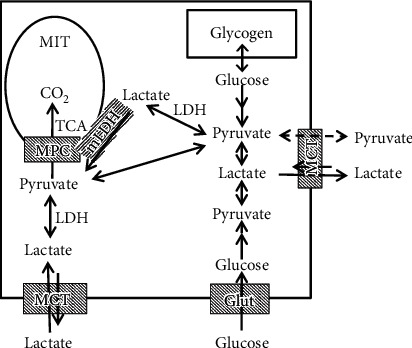
Schematic diagram showing the processes involved in cellular lactate biochemistry. The cellular lactate can diffuse in from the blood through the plasma membrane monocarboxylate transporters (MCT) or be produced from the reduction of pyruvate to lactate, catalyzed by the cytosolic LDH or mitochondrial mLDH. Pyruvate is produced by glycolysis of cellular glucose, which is derived from transport from the blood by the glucose transporter (GLUT) or produced from breakdown of intracellular glycogen (glycogenolysis). Pyruvate is transported into the mitochondria (MIT) via the mitochondrial pyruvate carrier (MPC) where it is oxidized to CO_2_. It is hypothesized that the mitochondrial oxidative lactate processes (left side) may be spatially separated from the glycolytic processes (right side), allowing simultaneous lactate uptake and release in the same cell.

Figure 1 schematically describes the physical relationships of the three lactate-related metabolic processes: (1) glycolysis (Eq. (1)), (2) pyruvate-lactate interconversion (Eq. (2), and (3) pyruvate oxidation in mitochondria. Although we will focus on the skeletal muscle cell, the diagram is applicable to other tissues, with the exception of the kidney and liver, which require the addition of a gluconeogenesis pathway. As the diagram illustrates, there are a variety of cellular locations where LDH catalyzes lactate-pyruvate interconversion. The metabolic pathways (arrows) in Figure 1 have been arranged to illustrate how a large fraction of the cellular carbohydrate metabolism might pass through lactate.

Both lactate and pyruvate are passively transported across the plasma membrane by the proton-linked monocarboxylate transporters (MCT) [20]. There are two isoforms (MCT1 and MCT4) present in the skeletal muscle. MCT1 has a higher Km (i.e., lower affinity) for lactate (4.5 mM) than pyruvate (0.7 mM) while MCT4 has a lower lactate Km (28 mM) than pyruvate (153 mM) [20]. MCT4 is located primarily in type II (anaerobic, glycolytic) muscle fibers [21] where its high pyruvate Km (low affinity) would, as desired, favor the export of lactate over pyruvate. In order to be oxidized, the pyruvate must be transported across the inner mitochondrial membrane, a function of the recently discovered “mitochondrial pyruvate carrier” (MPC) [22–24]. (The outer mitochondrial membrane is relatively freely permeable to molecules of the size of pyruvate [25].) Although knockout of MPC has dramatic effects on metabolism, pyruvate can still be oxidized via transamination to alanine which is then imported to the mitochondria [26].

The possible existence of a mitochondrial MCT [27] led to the suggestion that, in addition to the interorgan lactate shuttle, there was also an “intracellular lactate shuttle” in which lactate was directly transported into the mitochondria and oxidized [19]. This idea seems to be ruled out by the recent discovery of MPC and the observation that knockout of MPC prevents the direct oxidation of pyruvate [26]. If lactate could be transported directly into mitochondria, knocking out MPC should have minimal effect. The current view is that only pyruvate (not lactate) can be transported across the inner membrane [8, 9]. This new result with MPC has led to a modified version of the “intracellular lactate shuttle” in which lactate diffuses across the outer mitochondrial membrane to the intermembrane space where it is converted to pyruvate by a variant of LDH (mLDH) attached to the outer surface of the inner mitochondrial membrane (Figure 1) [8, 9]. Conditions in the intermembrane space putatively favor the lactate ➔ pyruvate reaction, producing a locally high pyruvate concentration that promotes rapid pyruvate transport across the inner membrane by MPC to the site of oxidation.

While most lactate-focused research groups favor the existence of mLDH [8, 9, 19], some studies have shown that isolated mitochondria oxidize pyruvate but not lactate; i.e., there is no mitochondrial conversion of lactate to pyruvate and mLDH is not present (see Ferguson et al. [9] for a detailed review). Evidence that this issue is not yet settled is illustrated by a 2019 publication that found no evidence for mLDH in either skeletal or cardiac muscle mitochondria [28]. One possible explanation for these discrepant results is that mLDH is sensitive to the mitochondrial isolation procedure. Although this is a highly contentious issue, it is not relevant to the question of a hypoxic versus nutritive model, which does not depend on whether the LDH is in the cytoplasm or the mitochondrial intermembrane space.

According to the “hypoxic” model, lactate is released from regions of the muscle cells which are anaerobic and cannot oxidize pyruvate. In contrast, according to the “nutritive” model, a significant fraction of the pyruvate is “purposively” converted to lactate and released to the circulation as an energy source for other tissues (the “lactate shuttle”). One of the main arguments for this “nutritive” model is the observation that there is simultaneous unidirectional tracer lactate uptake and release in the same muscle tissue (see Section 6). The favored interpretation of these simultaneous fluxes, shown diagrammatically in Figure 1, is that the lactate uptake from the blood is occurring in the “oxidative” (mitochondrial) region (Figure 1, left side) with release to the blood from the “glycolytic” region (Figure 1, right side) where glycogenolysis and glycolysis are producing pyruvate/lactate in excess of the rate these compounds are being oxidized; hence, there is abundant lactate available for export to other organs [29]. Strong arguments against this spatial separation of the glycolytic and oxidative regions are presented below.

## 3. Lactate-Pyruvate Exchange

LDH catalyzes a reversible exchange (Eq. (2)) in which lactate converts to pyruvate and vice versa. This has the potential to create major, not fully appreciated, problems with the interpretation of flux measurements using labeled lactate. For example, if this exchange rate was much greater than the rate of pyruvate metabolism, the tracer organ lactate uptake would simply be tracing pyruvate flux (Figure 1) and would not provide any information about net lactate metabolism [17].

While there is no doubt that such lactate-pyruvate exchange occurs, controversy exists with regard to the extent to which it accounts for the findings of studies utilizing labeled lactate to assess turnover. At one extreme, Sahlin [30] argues that the findings of labeled lactate studies simply reflect a high rate of LDH catalyzed lactate-pyruvate exchange (“nonproductive exchange” as termed by Landau and Wahren [31]) rather than turnover resulting from distinct metabolic reactions. A clear example of the dominance of such exchange over lactate metabolism is provided by in vitro studies of the fate of [^13^C]lactate added to a plasma suspension of erythrocytes. Since RBCs lack mitochondria, there can be no oxidation of the lactate formed via glycolysis; thus, total lactate production can be directly measured by the consumption of glucose. There was nearly complete [^13^C]lactate-pyruvate equilibration within 3 minutes (no equilibration occurred in the absence of the LDH provided by red cells) [32, 33]. The rate of erythrocyte lactate-pyruvate interchange was five times greater than the production rate of lactate; hence, labeled lactate flux measurements would massively overestimate the true lactate turnover rate of erythrocytes. Wolfe et al. [34] measured the ratio of dog plasma lactate/pyruvate ^13^C enrichment following a constant infusion of ^13^C-lactate and found a pyruvate enrichment within three minutes that was 92% of lactate, indicating that blood lactate and pyruvate also nearly equilibrate in circulating blood. Thus, in studies of lactate turnover, tissue is being perfused with labeled lactate and pyruvate of near equal specific activity. Based on this rapid lactate-pyruvate exchange, Wolfe and colleagues have used labeled lactate as a tracer of cellular pyruvate metabolism in a series of studies [35–38]. Further direct qualitative support for rapid exchange in tissues is provided by recent dynamic transfer measurements of hyperpolarized ^13^C exchange between pyruvate and lactate in tumor cells which showed that the labeling of lactate primarily results from exchange rather than net conversion of pyruvate to lactate [39, 40].

In one of the most influential recent publications in this field, Hui et al. [17] carried out a detailed analysis of the tracer exchange of glucose and lactate in the mouse. They found a basal lactate circulatory turnover flux of 374 *μ*mole/min/kg mouse, 2.5 times greater than the glucose turnover (1.25 times glucose on an absolute per g basis). About 32% of the labeled lactate appeared in serum glucose (i.e., gluconeogenesis), and 60% of the labeled glucose appeared in serum lactate. They estimated the contribution of lactate versus glucose to cellular oxidative mitochondrial metabolism in different organs by determining the steady-state relative tracer labeling of tricarboxylic acid cycle (TCA) intermediates. They concluded that, with the exception of the brain, more than 75% of the tracer-infused glucose passed through lactate (i.e., by interconversion with pyruvate, Eq. (2)) before being oxidized in the different body organs. They argue that these tracer fluxes represent distinct metabolic pathways (rather than exchange) and thus provide support for the “nutritive” lactate model. Their analysis of the kinetics of lactate-pyruvate exchange showed that even if “infinitely” fast, it could only equalize the lactate and glucose turnover (i.e., lactate is tracing pyruvate as discussed above). Since they found a glucose flux 1.25 times that of lactate, they claimed this argued against the exchange mechanism. However, by this argument, the exchange could still account for 1/1.25 or 80% of the tracer flux.

As discussed in Whole Body Circulatory Lactate Pharmacokinetics, two different approaches have been used to measure the human circulatory steady-state lactate turnover: (1) [^13^C] lactate tracer circulatory turnover and (2) the pharmacokinetics of a bolus infusion of nonlabeled lactate. If exchange were rapid relative to true turnover, tracer turnover would be appreciably greater than nonlabeled turnover. Based on the data shown in Table 1, the mean normal lactate turnover reported using the labeled and unlabeled techniques were, respectively, 17.2 and 11.3 *μ*m/kg/min (*p* < 0.036); i.e., the labeled technique yielded a mean turnover value that was 52% greater than that observed with the unlabeled lactate. The major potential error of the unlabeled technique is that infusion of exogenous lactate could slow the basal endogenous lactate production rate. Since this source of error artifactually increases the observed turnover rate, “correction” for this putative error would further increase the 52% difference between the labeled and unlabeled techniques.

It should be noted that in some tissues, e.g., brain, glucose is metabolized directly to TCA intermediates without exchanging with lactate [17], indicating that the importance of rapid lactate-pyruvate exchange may be variable depending upon the tissue. An argument against nearly instantaneous exchange is that tissue pyruvate tracer enrichment in skeletal muscle and GI tissue is only about 60% of the input lactate enrichment [32, 35].

Since many of the claims regarding lactate turnover (total circulatory and organ specific) are based on labeled lactate flux measurements, an understanding of these claims requires appreciation of the reported fluxes. Thus, this review of the literature will include data based on one-way flux measurements, but the reader needs to keep in mind that these fluxes potentially represent major overestimates of the true, metabolic fluxes.

## 4. The Krogh Model and Heterogeneity of Tissue O_2_ Partial Pressure

**Figure 2:**
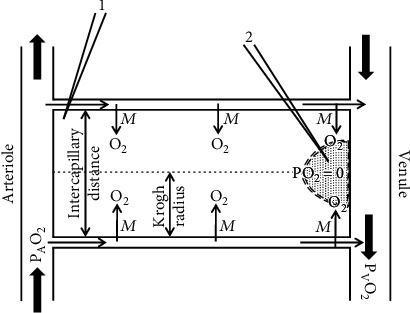
Diagram of the microcirculatory supply of oxygen (O_2_) to the tissue and the Krogh model of the local regulation of capillary blood flow. As the blood travels down the capillary, the O_2_ partial pressure falls from the arterial (P_A_O_2_) to the venous (P_V_O_2_) value. The PO_2_ falls as it diffuses from the capillary into the tissue as a result of the tissue oxygen consumption at the constant rate *M*. Krogh hypothesized that there was a region (dotted) at the venous end of the tissue where the PO_2_ fell to zero and that this hypoxic region produced a negative feedback signal that locally controlled the capillary flow. The position of the two O_2_ microelectrodes (labeled “1” and “2”) refers to the measurements of Whalen and colleagues discussed in the text.

The current lactate literature largely neglects one of the major triumphs of classical physiology: the Krogh model of microcirculatory blood flow regulation [41–43].  Figure 2 shows Krogh's idealized model of O_2_ delivery by the microcirculation. As the blood passes down the capillary, O_2_ diffuses into the tissue and is metabolized at a constant rate *M* per unit volume and the capillary pO_2_ falls from the arterial (P_A_O_2_) to the venous (P_V_O_2_) value. At the venous end, with the low driving force for O_2_ diffusion, a tissue region may become anoxic, indicated by the dotted area, because the O_2_ is used up before it reaches the center. Krogh's insight was that the rate of capillary blood flow is controlled locally by the requirement of minimizing the area of anoxic tissue. That is, the anoxic tissue region produces some signal that vasodilates the local arteriole supplying the capillary, setting up a negative feedback controlling capillary flow. Using just these assumptions along with the measurement of tissue O_2_ consumption (*M*), Krogh quantitatively predicted the intercapillary distance in the skeletal and cardiac muscle. This is one of the most dramatic examples of a theoretical prediction of an important physiological or biochemical parameter. Krogh [41] also showed that as the level of tissue activity (i.e., O_2_ consumption) decreased, the fraction of open capillaries decreased, effectively increasing the intercapillary distance, and the same control mechanism applied. Krogh received the Nobel Prize in 1920 for this work.

Although some aspects are still uncertain, most notably the identity of the anoxic signal [44], the main elements of Krogh's hypothesis are now generally accepted: that microcirculatory blood flow is locally regulated and that O_2_ delivery is the controlling factor. Despite this, the lactate literature emphatically states that in most conditions, there is no tissue region sufficiently anoxic to account for the tissue lactate production [8, 9]. This argument is based on a variety of measurements that indicate that the tissue pO_2_ is greater than the 1 mm Hg threshold required for optimal mitochondrial oxidative metabolism [9]. These measurements include the spectroscopic measurement of the NAD^+^/NADH ratio [45] and measurement of myoglobin saturation using either proton NMR [46, 47] or flash-frozen samples [48–50]. It is important to recognize that, as shown in Figure 2, muscle cellular pO_2_ is highly heterogeneous, with the anoxic tissue localized to a region in the center of the cell at the venous end. It would be necessary to sample a region less than 10 microns in diameter in order to detect the anoxia. Although it was originally claimed that the frozen myoglobin method had a resolution in this range [49, 50], this was later retracted [51]. It is now admitted that none of these techniques have sufficient resolution to detect single-cell pO_2_ heterogeneity and all such measurement reflect the average cellular pO_2_ [51]. With the regions of the cell at the arterial end having a pO_2_ of 50 mmHg or more [52–55], it would be nearly impossible for the average pO_2_ to be less than the 1 mmHg indicative of hypoxia.

Surprisingly, the lactate literature ignores the one method that does have sufficient resolution to identify small areas of hypoxia within a cell. Whalen and colleagues [52–55] published a series of articles in the 1970s describing the pO_2_ heterogeneity in the skeletal and myocardial muscle determined using pO_2_ microelectrodes with a 1- to 3-micron tip diameter. They impaled muscle with these electrodes and sampled random positions in the cell. They found, as predicted by Krogh, that the muscle tissue pO_2_ was extremely heterogeneous, ranging from 70 mmHg (presumably from an area of the cell near the arteriole, electrode no. 1 in Figure 2), down to 0 (electrode no. 2 in Figure 2). The histograms of the pO_2_ were in rough quantitative agreement with the prediction of the Krogh model. In both gracilis skeletal muscle and beating heart muscle, 50% or more of the pO_2_ measurements were in the 0-5 mm range [53].

An essential aspect of the Krogh model is that under all physiological conditions, some regions of the tissue must be anoxic in order to locally control blood flow. It is reasonable to assume that the anoxic regions would be net lactate producers, and this cellular pO_2_ heterogeneity could partially explain the simultaneous cellular uptake and release of lactate. The magnitude of the lactate production depends on the size of the anoxic region required for the successful feedback control of the microcirculation. Although the pO_2_ microelectrode measurements show large fractions (≈50%) in the 0-5 mmHg range, it is not clear what fractions of these measurements are less than the 1 mmHg pO_2_ threshold required for optimal mitochondrial oxidation. When anesthetized dogs are made hypoxic by ventilating with 8% O_2_ (arterial pO_2_ ≈ 28 mmHg), presumably increasing the size of the anoxic region, net muscle lactate release increases 18-fold, from 1 to 18 *μ*mol/min/kg dog [56]. As we will discuss below, this same mechanism of lactate release may contribute to the high blood lactate seen in exercise and shock.

## 5. Whole-Body Circulatory Lactate Pharmacokinetics

At steady state, the rates of lactate uptake (*Q*) from and release (*R*) to the circulating blood are equal. We will refer to this rate as the ^“^circulatory turnover flux^”^ = *R* = *Q*. Given that most conclusions regarding lactate physiology are based on this parameter (which is measurable in patients with shock), we have devoted this separate section to summarizing the available results (Table 1). Both unlabeled and tracer methods have been used, and, as discussed above, because the tracer methods may have a large artifactual “exchange” component, it is important to distinguish between them.

Table 1 summarizes most of the published whole-body human lactate circulatory measurements, and Table 2 lists a representative set of pharmacokinetic data based on this experimental data. There is a relatively large range in the reported tracer measurements of the normal basal circulatory turnover rate of lactate (*Q*), ranging from 11 to 28 *μ*mole/min/kg body wt., with an average value of 17.1 *μ*mole/min/kg body wt. [57–64]. There does not seem to be any consistent methodological explanation for this variability [63]. The tracer measurements of *Q* are about 52% greater than the nonlabeled measurements. As discussed above, this is what would be predicted if there was significant lactate-pyruvate exchange. For simplification, in this review, we will assume a basal *Q* value of 15 *μ*mole/min/kg (1.05 mmole/min/70 kg) (Table 2). Assuming a normal plasma lactate (*C*_p_) of 0.7 mM., steady-state lactate clearance (Cl_ss_ = *Q*/*C*_p_) is an impressive 1.5 L/min/70 kg, about 1/3 the maximum possible clearance of 5 L/min (i.e., the cardiac output of a 70 kg resting human). Note that in this review, we will not distinguish between “blood” and “plasma” lactate (both are used in the experimental literature). The blood lactate is about 20% less than plasma [65] because the steady-state erythrocyte lactate concentration is about half the plasma [65].

Several of the tracer lactate studies also simultaneously determined glucose pharmacokinetics, including the fractions of glucose and lactate that are oxidized to CO_2_ and the interchange of carbon atoms between lactate and glucose (see Tables 1 and 2) [57, 60, 66]. The glucose turnover is about 10 *μ*mole/min/kg, 33% higher than lactate turnover per gram of solute. The fraction of the lactate circulatory turnover that is oxidized to CO_2_ ranges from 0.45 to 0.85 in various reports [57, 59–61, 64, 66], and we will assume a value of 0.7 for both lactate and glucose (Tables 1 and 2). Thus, about 90 and 120 g/day/70 kg of lactate and glucose are metabolized, respectively (tracer measurements). To the extent that calculations based on studies with the labeled compound accurately reflect lactate metabolism, lactate is a major metabolite in humans, representing about 23% of the total basal metabolic rate of 1600 cal/day/70 kg.

Only about 15% of the lactate is converted to glucose in normal subjects (Table 1), in contradiction to the classic Cori model in which most of lactate is recycled back to glucose in the liver. This small fraction of conversion to glucose is predicted on the basis of energetics in that it is far more efficient for tissues to directly oxidize lactate than to convert it to glucose, a process that requires 6 ATP versus the 2 ATP released in the reverse, glucose to lactate, reaction [11]. The energetics of oxidizing glucose directly to CO_2_ via pyruvate is identical to that of cycling the glucose through lactate and then back to pyruvate to CO_2_. In contrast, glycogen synthesis (glycogenesis) is more efficient using circulating glucose, as opposed to lactate which must first be converted to glucose (using 6 ATP) before synthesizing the glycogen. From these considerations, one would predict that in Figure 1, the blood glucose would preferably be directed to glycogenesis and the blood lactate to oxidation.

The lactate volume of distribution (*V*_ss_) ranges from 35% of body weight, estimated from bolus nontracer lactate kinetics [67], to 49% when determined from the initial tracer dilution [61]. Assuming total body water and extracellular water are 60% and 25% of body weight, respectively [68], the corresponding value of the average tissue/blood lactate concentration ratio ranges from 0.29 to 0.69 for the 35% and 49% *V*_ss_ values. These estimates are in the same range as those determined from direct tissue measurements [69]. If lactate distributed as a univalent anion, then one would predict a tissue/blood ratio of about 0.1 because of the cellular membrane potential of 70 mV, inside negative. However, this assumption is incorrect because lactate is rapidly transported across cell membranes by the proton-coupled monocarboxylate transporters (MCT) as H^+^-lactate^−^ [69, 70] and this would be expected to produce an equilibrium tissue/blood ratio equal to [H^+^]_p_/[H^+^]_cell_ [71]. Since the average cellular pH is about 7.1 [72], this would correspond to a tissue/blood ratio = 0.45, similar to the experimental value. Assuming a *V*_ss_ value of 45% of body weight, one can estimate that the body pool of lactate has a basal turnover time (*T* = *V*_ss_/Cl_ss_) of about 21 minutes (Table 2).

**Figure 3:**
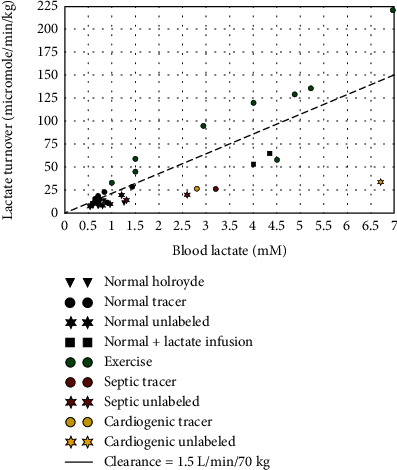
Summary of measurements of lactate turnover versus blood lactate in humans. The black points represent normal controls, without (circles or triangles) or with lactate infusion (squares or stars) to increase the blood concentration. The lactate turnover was measured using either the tracer (triangle, circle, and square) or unlabeled bolus (star) method. The data from Holroyde et al. (triangles) are individual subjects while all the other points represent the averaged reported results. The red and yellow points are for septic and cardiogenic shock, respectively, measured using either the tracer (circles) or unlabeled bolus (stars) method. The green points are for various levels of exercise. The dashed line shows the predicted relationship if the clearance has a constant value of 1.5 L/min/70 kg. Points above the line have a greater than normal clearance, and points below the line a less than normal clearance. See the main text for references.

As discussed above, the normal human lactate clearance (Cl_ss_) is about 1.5 L/min/70 kg at *C*_p_ = 0.7 mM. For most metabolic reactions, e.g., drug metabolism, the clearance mechanism does not saturate and hence remains constant as the solute concentration is increased; i.e., if the solute concentration is doubled, *Q* is also doubled. The relationship of lactate clearance to the blood lactate concentration in normal and shock subjects is of importance to differentiate between increased lactate production versus decreased lactate removal in the genesis of the increased blood lactate concentrations. Holroyde et al. [73] found that, in normal subjects, *Q* was linearly dependent on *C*_p_ over its normal range (0.5 to 1.25 mM), implying that the normal variations in blood lactate result from variations in the rate of lactate production rather than clearance. However, when blood lactate concentration was increased above the normal range via a lactate infusion, clearance decreased.  Figure 3 shows a scatter plot of the human *Q* versus *C*_p_ that summarizes all the data in Table 1. Plotted are the individual Holroyde et al. [73] data and the averaged data for normal subjects in whom *C*_p_ was increased by lactate infusion, along with measurements obtained during exercise and septic and cardiogenic shock. The dashed line in Figure 3 is the predicted relationship for a fixed clearance of 1.5 L/min/70 kg. It can be seen that the two data points (solid black squares) for normal subjects with high lactate concentration as a result of lactate infusions lie appreciably below the dashed line; i.e., clearance decreases as lactate catabolism saturates at high concentration. The clearance is reduced even further in the shock patients (yellow and red points). In contrast, the exercise data points lie above the dashed line, indicating that the lactate clearance is increased in exercise.

**Figure 4:**
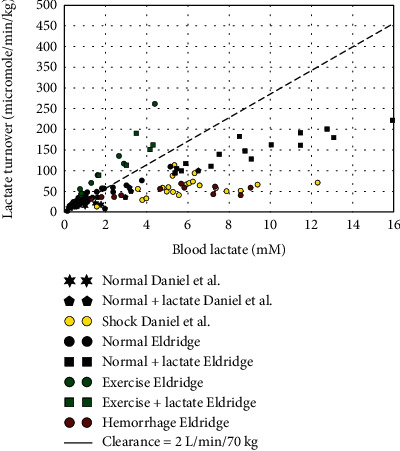
Summary of measurements of lactate turnover versus blood lactate in dogs reported by Daniel et al. and Eldridge (see main text for references). All of the points represent individual dogs with the turnover estimated using the steady state tracer method. The black points represent normal controls, without (circles or stars) or with lactate infusion (squares or pentagons) to increase the blood concentration. The yellow points are the measurements of Daniel et al. for septic, cardiac tamponade, and hemorrhagic-induced shock, and the red points are the hemorrhagic shock results of Eldridge. The green points are for various levels of exercise, either without (circles) or with (squares) lactate infused to increase the blood concentration. The dashed line shows the predicted relationship if the clearance has a constant value of 2.0 L/min/70 kg.

Figure 4 shows a similar plot of *Q* versus blood lactate in dogs. The points represent the individual measurements of Daniel et al. [74] and Eldridge [75–77]. The black points show data for normal dogs, with or without lactate infusion; the green and red points are the exercising and hemorrhagic shock data of Eldridge; and the yellow points are the results of Daniel et al. [74] for hemorrhagic, endotoxin, or cardiac tamponade-induced shock. The dashed line shows the predicted result for a clearance of 2.0 L/min/70 kg, the value observed in unmanipulated dogs. Of importance, the lactate turnover in normal dogs (solid squares) levels off for plasma concentrations above 8 mM, as the lactate disposal rate becomes saturated. As a result, the usual homeostatic response to a rising blood solute level is no longer operative, and blood lactate concentration will continually increase as long as lactate production exceeds the saturated removal rate. Induction of shock further reduces the clearance observed with high blood lactate levels, while clearance is increased in exercise. These results are discussed in more detail in Exercise, Septic Shock, and Hemorrhagic and Cardiogenic Shock.

## 6. Lactate Uptake and Release by Individual Organs

Understanding the contribution of various organs to the circulating lactate is complicated by the observation that organs appear to simultaneously release and take up lactate. As discussed above, with a homeostatic blood lactate of 0.7 mM and a lactate clearance of 1.5 L/min/70 kg, at steady state, there is an equal rate of lactate uptake from and release to the blood of 1.05 mmole/min/kg, a value known as the “circulatory turnover” of lactate. The contribution of various organs to this turnover is determined by the unidirectional rates of organ release (*R*) and uptake (*Q*), which are directly measured from A-V differences of tracer and unlabeled lactate concentrations across the organs following achievement of a steady state for the tracer blood concentration during a constant tracer infusion. As discussed above, the underlying mechanism(s) responsible for the organ tracer fluxes are controversial. In this section, we will make the conventional assumption that these simultaneous fluxes measure the distinct metabolic uptake (*Q*) and release (*R*) processes. The experimental results of these measurements for the major organs in normal humans are summarized in Table 3 (see the table for references). Unidirectional organ tracer fluxes are available only for the heart, brain, and skeletal muscle.

Although the liver plays a central role in regulating blood lactate, no A-V difference measurements are available in healthy humans because of the difficulty of sampling portal vein blood. There have, however, been a large number of reports of the normal human resting postprandial splanchnic (gut plus liver) A-V difference [78–82] determined from arterial-hepatic vein lactate concentration difference, all indicating that there is net splanchnic lactate uptake. The reported uptake values have a very wide normal range, varying from 0.01 [82] to 0.48 [80] mmole/min/70 kg, and we will assume a mean value for the splanchnic uptake of lactate of 0.2 mmole/min. Simultaneous measurements of the gut (artery–portal vein) and liver (portal vein + hepatic artery–hepatic vein) A-V differences in the dog show that the liver's handling of lactate varies in response to physiological conditions [83–85]. Following a meal, there is an initial (0 to 6 hours), large hepatic lactate output of 10 to 15 *μ*mole/min/kg dog, which produces, at peak, a nearly 3-fold increase in blood lactate [83]. This changes to a net hepatic uptake 20 hours after the meal, reaching 5 *μ*mole/min/kg at 30 hours [83]. Over the same period, the gut has a relatively unvarying lactate output of about 2 *μ*mole/min/kg dog [83]. During prolonged exercise, the dog hepatic lactate uptake rapidly increased from the resting value of 3.9 to 25 *μ*mole/min/kg and then slowly decreased, becoming a net lactate output of about 11 *μ*mole/min/kg after 160 minutes of exercise [85]. Portal glucagon infusion produces an initial net lactate output that becomes a net uptake after about 3 hours [84]. Given these large physiological variations in dog liver lactate extraction, it is not surprising that the human splanchnic extraction has such a large “normal” range.

In Table 3, we have extrapolated the dog gut data to the human [86], yielding a net gut release (*R*) of 0.08 mmole/min/kg. Given this gut release, liver net uptake must be on the order of 0.28 mmole/min to yield the observed splanchnic uptake 0.20 mmol/min. Since there are no tracer lactate measurements of unidirectional fluxes, these represent minimum contributions to circulatory turnover.

One definite source of lactate production is erythrocytes, which have only glycolytic metabolism. At physiological pH, erythrocytes consume about 1.5 mmole glucose/L cells/hr [87]. Since each glucose consumed produces 2 lactates, this corresponds to a total body lactate production of 0.105 mmole/min, assuming 2 L cells/human (Table 3).

In the steady state, the total tracer uptake and release should be equal to each other and equal to the independently measured circulatory turnover ( = Cl_ss_∗C_p_ = 1.05 mmole/min/70 kg, Table 2) discussed in Whole-Body Circulatory Lactate Pharmacokinetics. It can be seen in Table 3 (bottom line) that this condition is approximately satisfied by the estimated values. This suggests that the “minimum” estimates in Table 3 are close to the correct values and there is insignificant simultaneous uptake and release in kidney, gut, liver and adipose tissue. Another possible source of exchange, not included in Table 1, is the lungs, which are difficult to quantitate because their high blood flow tends to obscure small A-V differences that could be significant when multiplied by the large blood flow [29].

## 7. Exercise

The increase in blood lactate noted during exercise initially prompted the historical interest in lactate, and the bulk of recent lactate research has been devoted to understanding the physiology of lactate during exercise. Some of the factors involved in the increase in blood lactate during exercise may also be applicable to shock. Historically, an increase in blood lactate during exercise had been regarded as an indication of systemic “dysoxia,” i.e., the inability of the circulation to meet the O_2_ requirements of the muscle (see Ferguson et al. [9] for details). In 1964, Wasserman et al. [88] proposed the term “anaerobic threshold” as a noninvasive marker of dysoxia defined as the exercise level when the respiratory exchange ratio $\left(R=\dot{\text{V}}\text{C}{\text{O}}_{2}/\dot{\text{V}}{\text{O}}_{2}\right)$ sharply increased as a result of bicarbonate-derived CO_2_ produced from the buffering of the increased blood lactic acid [89]. More recent definitions of this threshold are more directly linked to blood lactate changes. At low to moderate levels of exercise, blood lactate first increases and then levels off or decreases, while at higher levels, it continues to rise. The “lactate threshold” (also referred to as “anaerobic threshold”) is defined as the maximum exercise level at which blood lactate does not continue to increase. The standard blood lactate corresponding to this threshold is about 4 mM, but there are large individual variations. Since the blood pyruvate only slightly increases during exercise, the blood lactate/pyruvate ratio increases from about 10 at rest to 30 or more at intense exercise levels [90]. These changes in blood lactate during exercise have been reinterpreted in the recent lactate literature, and the current dominant view is that they are explained primarily in terms of a “shuttling” of lactate as a means of redistributing an energy source with little or no relation to anoxia [9, 91].

**Figure 5:**
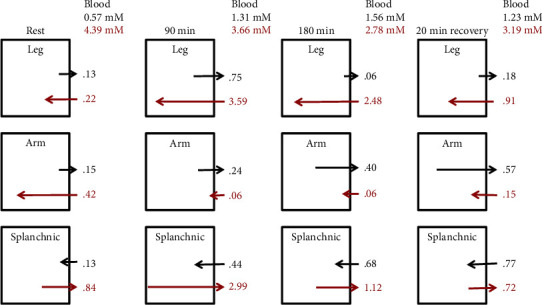
Measurements by Ahlborg et al. of the leg, forearm, and net splanchnic lactate (black) and glucose (red) fluxes and blood concentrations at rest and at 90 and 180 minutes of moderate bicycle exercise and after a 20-minute recovery. The fluxes are in units of mmole/min.

Lactate and glucose metabolism has a complex dependence on the details and intensity of the exercise protocol. This will be illustrated by the three following examples of lactate measurements during increasing exercise intensity.  Figure 5 diagrams the forearm, leg, and splanchnic net lactate and glucose exchange (in units of mmole/min) over a long time period (180 minutes) of moderate (58% of maximum VO_2_) exercise on a bicycle ergometer described by Ahlborg and Felig [92]. The blood lactate and glucose concentrations and net fluxes (measured via A-V differences) are shown at rest and at 90 and 180 minutes of exercise and 20 minutes after recovery. At this relatively low level of exercise, the blood lactate approximately doubled and was relatively constant throughout the exercise and recovery period; i.e., this was below the “anaerobic threshold.” The blood glucose fell continuously, reaching hypoglycemic levels (2.78 mM) by 3 hours. There was a small resting net lactate efflux in the leg, which increased 5.7-fold after 90 minutes of exercise but then returned close to the resting value after 180 minutes of exercise and during recovery. The lactate release was small compared to the glucose uptake (red arrows) which increased by 15- and 11-fold at 90 min and 180 min, respectively. Thus, at this exercise level, circulating lactate did not provide a net carbohydrate source for the exercising muscle because there was a small net muscle lactate release. Surprisingly, during the experiment, there was a continuous increase in lactate efflux from the arm (which was not exercising), which appreciably exceeded that of the leg at 180 minutes of exercise and after 20 minutes of rest. Since this lactate release was greater than could be explained by glucose uptake, it is necessary to postulate that leg exercise somehow activated the breakdown of arm muscle glycogen, possibly as a result of the hypoglycemia. The splanchnic (presumably liver) lactate uptake increased about 4-fold during the exercise, roughly balancing the arm and leg release. The splanchnic glucose release increased 3.5-fold after 90 minutes and then decreased as the exercise was prolonged, presumably due to depletion of liver glycogen. During the recovery period, the splanchnic net lactate uptake became greater than the glucose release.

Van Hall et al. [93] have carried out what is probably the most detailed study of the tracer lactate and glucose fluxes during exercise. They infused labeled lactate, glucose, and glycerol and determined whole-body, leg (femoral vein sample), and arm (subclavian vein) lactate uptake (*Q*) and release (*R*). The measurements were made at rest and then during a 40 min period of the classical diagonal arm+leg cross-country skiing technique, followed immediately by a 10 min double arm poling (no leg activity). The arm+leg phase is at a relatively moderate exercise level (for these elite cross-country skiers) that can be maintained for a long time, while the arm poling is more intense, leading to fatigue.

**Figure 6:**
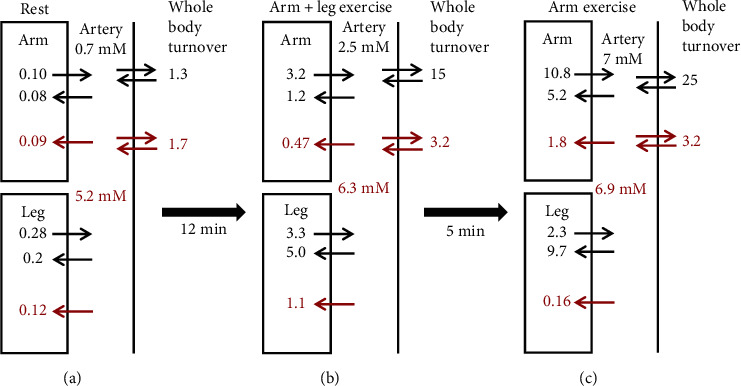
Diagrammatic representation of experimental measurements of Van Hall et al. The arm and leg unidirectional lactate (black arrows) and glucose (red arrows) fluxes (in units of mmole/min/70 kg) at rest (a) and during moderate arm and leg exercise (b) followed by exhaustive arm exercise (c) are indicated. Also shown are the whole-body lactate turnover (black) and glucose (red) turnover. The corresponding arterial blood lactate (black) and glucose (red) concentrations are also indicated. The arm fluxes and whole-body turnover are shown in the top panels and the leg fluxes in the bottom panels. The time intervals separating the three experimental exercise states are also shown.

Figure 6 summarizes the total circulatory, arm, and leg unidirectional lactate (black) and glucose (red) fluxes (in units of mmole/min) (the net flux is the difference between these fluxes). The arterial plasma lactate was 0.7 mM at rest and rose to about 2.5 mM after 12 minutes of arm+leg skiing and remained at that level through 40 minutes (i.e., below the anaerobic threshold) and then during the arm poling rose very rapidly (i.e., above the anaerobic threshold), within 5 minutes, to about 7 mM (the same as the blood glucose). Interestingly, during the moderate arm+leg phase, there was a net lactate uptake in the exercising legs, which seemingly demonstrates that circulating lactate can provide an energy source for exercising muscle. This differs from the results of Ahlborg et al. (Figure 5) when at no time was there net muscle lactate uptake. This difference probably is due to the much higher blood lactate (about 10 times normal), which provides a larger driving force for muscle lactate uptake. The rapid rise in the blood lactate during the arm exercise presumably resulted from the dramatic increase in net arm release of 5.6 mmole/min/70 kg (280 times the resting arm value) at this exhaustive (i.e., glycolytic) arm workload. Note that the leg lactate uptake nearly balances this arm lactate release and is more important than the liver in clearing lactate in this period. During the arm exercise period, when the legs were recovering from exercise, the net leg lactate uptake was dramatically increased to 7.4 mmole/min/70 kg, while the leg glucose uptake was reduced to near zero. It is important to emphasize that it is essential for the validity of these tracer flux measurements that the labeled and unlabeled blood and exercising muscle lactate concentration is in a steady state. Clearly, during exercise, when the concentrations are changing over a period of minutes, these flux values are only rough estimates [93].

Van Hall et al. [93] also measured (Figure 6(c), right side) the whole-body circulatory lactate (black) and glucose (red) turnover (*Q*). At rest, *Q* was 1.3 mmol/min/70 kg and increased 11-fold to 15 mmol/min/70 kg within 12 minutes of arm+leg poling and then increased further to 25 mmol/min/70 kg within 5 minutes of arm poling (see Figure 6). The whole-body glucose turnover roughly doubled during the two exercise phases and was only about 1/8 of the whole-body lactate turnover during the exhaustive arm exercise. They also determined the amount of lactate that was oxidized (appeared as CO_2_) for the arm, leg, and whole body. About 40% of the lactate turnover was oxidized at rest, increasing to nearly 100% during the arm+leg exercise. At rest, lactate oxidation represented about 30% of the carbohydrate metabolism with glucose oxidation contributing the remaining 70%. The whole-body O_2_ uptake (i.e., energy consumption) increased about 10-fold during exercise. Because the lactate turnover increased ≈15-fold, its contribution to carbohydrate metabolism remained at about 30%. In contrast, because glucose turnover only doubled during exercise (Figure 6), the glucose component of carbohydrate metabolism decreased to about 12%, with the remaining 58% supplied by glycogen breakdown. Thus, during exercise, the circulating blood lactate metabolism contributes more than twice as much energy as the blood glucose.

**Figure 7:**
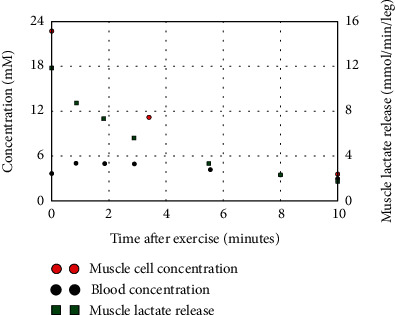
Plot of the blood (black circles) and muscle cell (red circles) lactate concentration (mM) and muscle lactate release (mmol/min/leg, green squares) following intensive leg exercise.

During intense exercise, the muscle plasma membrane MCT lactate transporter becomes rate limiting, resulting in large tissue/plasma lactate gradients. Karlsson and Saltin [94] measured blood and muscle tissue lactate in subjects at intense bicycle workloads, leading to exhaustion at 2-3 minutes. At the end of exercise, the muscle lactate had risen about 16-fold to 16 mM, while the blood lactate was only 7.4 mM. During recovery, the blood lactate continued to rise as the lactate diffused out of the muscle, reaching a peak value 13.5 mM.  Figure 7 shows a plot of the measurements of Bangsbo et al. [95] of muscle and blood lactate and net muscle lactate release following intensive one-leg exercise that produced exhaustion at 3.5 minutes. At the end of the 3.5 minutes of exercise, the muscle lactate (red circles) has risen 22-fold to 22 mM while the blood lactate (black circles) is about 3.7 mM. The net muscle lactate release (green squares) increased from near zero at rest to 12 mmol/min/leg at the end of exercise (time = 0, Figure 7). This release rate from one leg is 12 times that of the whole-body resting circulatory turnover of 1.05 mmol/min/70 kg (Table 2). The rate of muscle lactate release falls back to near zero after 10 minutes as the lactate diffuses out of the cell and the cell concentration falls to that of the blood lactate (=3 mM).

These results clearly illustrate that there can be “shuttling” of lactate between organs (i.e., arm, leg, and liver) during exercise and that, above the anaerobic threshold when there is high blood lactate, lactate can become a primary energy source for tissues other than the liver. However, these results do not provide direct support for the central idea of the “nutritive model” that the lactate release is not simply the result of muscle hypoxia but rather is an advantageous adaptation. Almost certainly, the dramatic increase in lactate efflux and blood lactate during intense exercise results from hypoxia, with the glycogenolysis (glycogen ➔ glucose ➔ lactate) providing an anaerobic energy source (Eq. (1)). The net leg lactate uptake by the less intensely exercising leg muscles (Figure 6) probably results from the mass action effect of the markedly increased (10-fold) blood lactate concentration. Thus, the lactate release in these exercise experiments seems to be consistent with an anoxic muscle mechanism, as discussed above in The Krogh Model and Heterogeneity of Tissue O_2_ Partial Pressure. The best support for a nonhypoxic lactate release is the increase in lactate release by the nonexercising arms after about 180 minutes of bicycling in the experiments of Ahlborg et al. [92] (Figure 5). This lactate was rapidly removed by the splanchnic bed, and it could be postulated that shuttling of lactate between the arm and splanchnic bed served a useful function by supplying substrate for glucose production via the Cori cycle to combat the hypoglycemia. However, the arm lactate release might have resulted from the hypoglycemia which, by itself, can produce increases in blood lactate [96].

## 8. Septic Shock

Shock is classically defined as a clinical state in which there is either absolute or relative tissue hypoperfusion and/or hypoxia [97]. There are two basic forms, distinguished by the cardiac output (CO). The first is when the initiating hypoxic event is decreased O_2_ delivery from a reduced CO, secondary to either hemorrhage [98] or cardiac dysfunction [99]. The second form is septic shock in which an intense inflammatory response leads to marked vasodilation and hypotension in the setting of an increased CO (see Table 4). Regional differences in vascular tone and microcirculatory dysfunction [100] and, in its extreme, microvascular thrombosis [101, 102] can lead to regional hypoperfusion and organ dysfunction (also referred to as vasodilatory or distributive shock). In both forms, the increase in blood lactate is strongly correlated with mortality. This review will focus primarily on septic shock which is the most common clinical form and where understanding the associated lactate biochemistry may help elucidate its pathophysiology.

The centrality of lactate in septic shock is illustrated by what has recently become its consensus definition: a “…vasopressor requirement to maintain arterial pressure of 65 mm Hg or greater and serum lactate level greater than 2 mM in the absence of hypovolemia” [103]. The septic shock literature commonly states that hyperlactatemia is a result of the combination of excessive production in muscle and defective lactate removal by the liver and kidney. However, as the subsequent discussion will demonstrate, these statements are not based on quantitative assessment of the flux in these organs. Quantitative measurement of circulatory turnover and organ fluxes requires the establishment of a steady state for infused labeled lactate—a process requiring several hours in healthy subjects. The paucity of quantitative data in septic shock no doubt reflects the difficulty of performing this arduous technique in these patients who commonly are, by definition, critically ill and undergoing intense resuscitation interventions.

Further complicating matters is that septic shock classically presents in two phases. The early phase prior to resuscitation is characterized by low CO due to a marked increase in venous capacitance lowering mean systemic pressure and mimicking hypovolemic shock. Only after the initial fluid resuscitation are filling pressures restored allowing for the high CO classically associated with the later phase of septic shock. This shift in underlying physiology may have important implications for the etiology and clinical relevance of increased blood lactate in different stages of sepsis. The few available human measurements have been obtained during the later stages of shock, i.e., after fluid resuscitation and administration of vasopressors (e.g., dopamine, norepinephrine, epinephrine, and vasopressin) [104], which no doubt alter the pathophysiology. Conversely, most animal models lack aggressive fluid and vasopressor support and may more closely model the early unresuscitated phase of septic shock (Table 4). The best data for the clinical relevance of hyperlactatemia relate to the strong positive correlation with survival in patients who “clear” their elevated lactate after initial resuscitation [105, 106]. This early “clearance” may as much reflect an increase in CO allowing more blood flow to the liver as it does resolution of hypoxic tissue beds. Because of these limitations, the following discussion primarily raises questions without providing definitive answers.

There are two markedly different explanations for the increased lactate in septic shock. The classical “hypoxic” model is that the lactate elevation is an indication of some poorly characterized local tissue hypoxia, with the increased blood lactate serving as a measure of the degree of this “occult hypoperfusion” [107, 108]. In response to the hypoxia, there is a vasodilation and increased blood flow, i.e., a decreased systemic vascular resistance (SVR) which is a central clinical feature in septic shock [109]. In contrast, proponents of the “nutritive model” propose that the increased lactate in septic shock is not an indication of hypoxia, but rather a sign of a stress response or “hypermetabolic state,” similar to exercise, in which the increased lactate putatively is providing a beneficial metabolic energy source [9, 11, 13].

When confronted with an elevated concentration of any serum analyte, the initial physiological question is whether the elevation results from an increased release into the blood versus decreased clearance. Surprisingly, only two studies in humans have attempted to answer this basic question—does the elevated blood lactate of septic shock result from increased delivery versus decreased clearance of lactate from blood—and these two studies yielded conflicting results.

Revelly et al. [60] used the steady-state tracer-labeled lactate infusion method to determine lactate uptake (*Q*) in both septic and cardiogenic shock patients treated with fluids and vasopressors. They found a roughly 3-fold increase in blood lactate and a small (≈20%) decrease in clearance in septic shock—i.e., most of the increase in blood lactate was the result of increased lactate release. Levraut et al. [110] determined lactate uptake using the bolus infusion of unlabeled lactate in mild septic shock patients who did not need vasopressors. They found a 2.17-fold increase in blood lactate and a 2.12-fold decrease in clearance—i.e., most of the increase in blood lactate was the result of decreased clearance. It is not clear if these dissimilar observations reflect different methodologies versus different clinical status of the patients.

The few measurements of CO or cardiac index (CI), splanchnic and muscle blood flow, and splanchnic lactate flux in patients purported to have human septic shock are summarized in Table 4. The CI is in units of the fraction of the normal human resting CI (=3.3 L/min/m^2^), and the organ flows are in units of the fraction of the corresponding CO. The near-normal blood lactate of most of these patients suggests they had a very mild version of septic shock or were well into recovery. CI in these treated septic shock patients is increased from 40 to 70%. The increase in CI is primarily a result of the splanchnic flow which increases to 30 to 47% of cardiac output from a normal value of about 20–25% [111, 112]. There is just one estimate of shock muscle flow of about 20% of CO [113], slightly greater than normal, based on extrapolating femoral artery blood flow to whole-body muscle (assuming lower body muscle is 56% of the whole body [114].

This large increase in splanchnic flow might be a vasodilatory response to gut hypoxia and an indication that the gut is the major organ affected by shock and the source of the increased lactate production. Unfortunately, there are no measurements of gut lactate flux in human shock. There are 3 measurements of human splanchnic flux (Table 4), all of which indicate a net lactate uptake. This could be consistent with the increased gut lactate release shock explanation since, as long as the splanchnic uptake is less than normal, it would produce an increased blood lactate. This gut hypoxia was reproduced experimentally in anesthetized pigs [115] in which the gut blood flow was selectively decreased by about 60% by shunting aortic blood past the mesenteric vessels, with all other flows remaining normal. This produced about a 3-fold increase in gut lactate release and an increase in arterial lactate from 0.8 to 3.2 mM, demonstrating that the blood lactate levels seen in shock can be produced solely by the gut. Although there was still a net splanchnic uptake, because this uptake was less than normal, it could have accounted for the increased blood lactate. The quantitative relationship between splanchnic uptake and blood lactate is discussed in more detail below (see Eq. (3)).

Although there are no direct measurements of human shock muscle lactate output, indirect evidence for increased production is provided by measurements of muscle tissue lactate concentration [116, 117]. In normal subjects, there was no significant difference between the muscle and blood concentration, while in patients with septic shock, muscle lactate was 2.9 mM greater than blood, consistent with increased muscle production [117].

An argument against the hypoxic model is that increasing tissue O_2_ delivery (DO_2_) is of no benefit in shock patients [13]. Although transfusing packed red cell increased DO_2_ by about 20%, it did not have any beneficial effect on septic shock patients as measured by blood lactate [118], splanchnic lactate uptake [118], systemic O_2_ uptake [119], or gastric intramucosal pH [119]. However, this argument ignores the obvious clinical benefit of fluid resuscitation in early sepsis and these experimental results should instead be interpreted as indicating that further increasing DO_2_ beyond already supranormal levels does not improve these resuscitation parameters. If, as discussed below, shock produces a microvascular defect that greatly increases the local tissue O_2_ diffusion distances [120], small increases in DO_2_ are unlikely to improve function.

The best controlled human data for a mild form of septic shock are those from two studies of the effects of E. coli endotoxin in normal volunteers that produce small increases in blood lactate (to about 1.2 mM) resulting from an increase in lactate production with no change in clearance [121, 122]. The endotoxin produced a doubling of splanchnic blood flow with no change in leg blood flow or muscle output which, again, suggests that the gut is the main source of this increased lactate production.

There are dramatic increases of arterial epinephrine (about 40-fold) and norepinephrine (about 8-fold) in septic shock, and there is a high correlation between blood norepinephrine levels and shock severity (e.g., nonsurvivors vs. survivors) [123]. Studies in normal human volunteers have shown that increasing blood epinephrine levels can duplicate some of the changes seen in lactate metabolism in shock. Clutter et al. [124] determined the effect of different rates of epinephrine infusion in normal volunteers. At the highest rates, which increased blood epinephrine about 50-fold, blood lactate increased from 0.85 to 2.56 mM, similar to the changes seen in mild septic shock. The proponents of the “nutritive” lactate model have suggested that the primary cause of increased blood lactate in shock is the high catecholamine levels that directly stimulate glycogenolysis and lactate release without any increase in hypoxia [11]. However, the observed increase in blood lactate could also result from an increase in hypoxic tissue as a result of the general thermogenic action of catecholamines, increasing O_2_ consumption by up to 35% [125, 126].

Although more detailed measurements are possible in animal models of septic shock (e.g., dog, pig, sheep, and guinea pig), there is major uncertainly about the relationship of these models to human septic shock. In most cases, the animals are anesthetized and treated only with fluid resuscitation (not vasopressors) and the procedures used to induce septic shock (e.g., endotoxin or peritonitis) may not be representative of human shock [127]. Daniel et al. [74] measured the whole-body lactate turnover in the dog following shock produced by endotoxin (i.e., septic), cardiac tamponade, or hemorrhage. The results were similar in all three shock forms, with about a 6-fold increase in blood lactate and a 3-fold increase in lactate turnover. These results are plotted in Figure 4 (yellow points). It can be seen that lactate clearance is about half of normal in all three forms of shock. However, the clinical relevance of this dog septic shock model is questionable since the metabolic rate is reduced, in contrast to the human where it is increased. Wolfe et al. [128] carried out a more detailed study of endotoxin shock in conscious dogs. Following a 2-minute intravenous endotoxin infusion, there was an initial large transient drop in blood pressure and CO that returned to near normal by 30 minutes, while there was a persistent 50% increase in heart rate. Within 30 minutes, the blood lactate increased 10-fold, from 0.7 to 7.0 mM, where it remained for 3 hours and, in the same time period, the lactate turnover increased about 3.4-fold, from 7 to ≈24 *μ*mol/min/kg. However, as discussed by the authors, these results may not be representative of human septic shock because the endotoxin induced a dramatic and persistent hypoglycemia which was associated with an increased glycogen breakdown and a slow increase in the percent of the glucose derived from lactate (from 20% to 80%). This suggests that the increased lactate turnover was secondary to the hypoglycemia, a factor which is usually not relevant in human septic shock where sepsis is often associated with insulin resistance and hyperglycemia.

Table 4 summarizes most of the data that are available for organ flux of lactate in animal models of septic shock. In general, the utility of these measurements is limited by their failure to simulate human septic shock. It can be seen that, with the exception of the sheep studies [129, 130], the models do not duplicate the large increase in CO seen in human septic shock, presumably because of the lack of the intense fluid and vasopressor resuscitation which characterizes the human shock state in clinical settings. Two of the studies show that the liver uptake of lactate increases roughly in proportion to blood lactate [131, 132], which would suggest a constant clearance, while the study of Tapia et al. [130] reports a dramatic decrease in clearance. One consistent observation of these animal shock models in Table 4 is that splanchnic blood flow in sepsis increased the same or more than the CO while muscle blood flow was constant or decreased, similar to what is observed in humans.

A theme that frequently resurfaces in the shock literature is that of a “mitochondrial dysfunction” as the cause of the increased lactate [14, 133, 134]. Not surprisingly in these severely ill shock patients, there are a variety of pathological changes that can be detected in mitochondrial morphology and function. However, it is unclear if these changes are sufficient to actually limit pyruvate oxidation and produce the increased lactate production observed in shock. A 2018 systematic review of the literature concluded that “… the current state of this evidence is limited to laboratory investigation, it remains to be tested in-vivo to determine the clinical significance for mitochondrial dysfunction as a manifestation of disease in sepsis” [135]. Mitochondrial dysfunction, if it were significant, could be regarded as another form of hypoxia (i.e., as a result of decreased oxidation but due to mitochondrial dysfunction instead of low pO2) and would be consistent with the hypoxic model of septic shock.

The proponents of the nutritive model have reasoned that septic shock, with its characteristic increased cardiac output, is a “hypermetabolic” state, similar to exercise, and the increase in blood lactate is produced by increased glycogenolysis in excess of pyruvate utilization in some organs that lead to the export of lactate as a circulating energy source for other organs. However, as is illustrated in Table 1 and Figures 3 and 4, shock (septic, cardiogenic, and hemorrhage) differs markedly from exercise where there is some evidence for such lactate shuttling. Although there is an increase in whole-body lactate turnover in shock, this increase is about half of what one would predict for normal subjects with the same increase in blood lactate and about one-fourth of that observed in exercise. That is, there is a decreased clearance in shock in contrast to the increased clearance in exercise. In addition, from the limited measurements that are available concerning individual organ blood flow and lactate flux in septic shock (Table 4), there is no significant increase in either muscle blood flow or lactate flux. Thus, there is no experimental support for the existence of the hypermetabolic state predicted by the nutritive model.

Finally, while multiple older studies of human sepsis reported a supply dependence to oxygen consumption (VO_2_), they likely were a result of compounded errors in measurement of both VO_2_ by the Fick equation and CO by thermodilution (i.e., errors in measured CO similarly impact both VO_2_ and DO_2_) creating a spurious linear correlation [136, 137]. Studies using independent measurements of VO_2_ by indirect colorimetry have found only mildly decreased to mildly increased VO_2_ without supply dependence after initial resuscitation [138], further arguing against sepsis being a hypermetabolic state.

## 9. Hemorrhagic and Cardiogenic Shock

These two forms of shock have in common a decreased CO, which is assumed to be the fundamental cause of the hypotension [99]. Cardiogenic shock patients have life-threatening heart failure and, at the time the physiological studies have been performed, intensive fluid and pharmacological (e.g., vasoconstrictor) therapies have been initiated. While decreased CO obviously must result in an overall decrease in tissue perfusion, we found no quantitative values in the literature as to how this decreased CO is distributed to the skeletal muscle and splanchnic tissues in human cardiogenic shock.

There are two reports of whole-body lactate turnover in human cardiogenic shock (Table 1). Chiolero et al. [139] compared the clearance determined using a transient infusion of unlabeled lactate in normal versus cardiogenic shock patients. The cardiogenic patients had an average blood lactate of 6.7 mM (7 times normal) and a slightly (25%) reduced lactate clearance. Similarly, Revelly et al. [60], using a steady-state tracer technique to measure lactate turnover in cardiogenic shock patients and normal controls, found that the patients had an average blood lactate of 2.8 mM (3.1 times normal) and a 20% reduction in lactate clearance. Thus, both studies found a modest dependence of clearance on blood lactate concentration, similar to what is seen in normal subjects when blood lactate is increased by lactate infusions [75]. These results are plotted in Figure 3 (yellow points) where the decrease in lactate clearance is clearly illustrated.

Human hemorrhagic or hypovolemic shock represents a heterogeneous range of conditions caused by a variety of events (most commonly trauma) that produces hypovolemia or blood loss [140, 141]. Depending on its severity, the resultant hypoxia produces a range of tissue pathologies (i.e., the shock state) that may be irreversible and persist even after the volume loss is corrected. The initial blood lactate before treatment is the best available marker for the severity of the trauma and the subsequent decease of the lactate with treatment is a good indicator of the patient's recovery and survival [142, 143].

Not surprisingly, there are no quantitative physiologic studies prior to fluid replacement or resuscitation of hemorrhagic shock in humans. However, this form of shock is the best studied and easiest form of shock to model in animals. Wiener and Spitzer [144] carried out a detailed investigation of lactate metabolism following severe hemorrhage (56% of blood volume) in conscious dogs using the steady-state tracer procedure to determine lactate turnover. The cardiac output fell by half, O_2_ consumption by 27%, and mean blood pressure by 47%. The arterial lactate increased more than 5-fold (from 1.27 to 6.54 mM), and the lactate turnover and the amount of lactate oxidized to CO_2_ both increased about 3-fold, with lactate supplying more than 50% of the oxidative substrate. The lactate clearance fell in half after the hemorrhage, indicating that both increased lactate production and decreased clearance were responsible for the elevation of the blood lactate. Eldridge [75] carried out a more detailed analysis of the change in clearance following varied amounts of hemorrhage in anesthetized dogs, again using steady tracer measurements of lactate turnover (data plotted in Figure 4, red circles). They also determined the dependence of the clearance on blood lactate in normal dogs by infusing unlabeled lactate to raise the blood concentration. As can be seen in Figure 4 (red points), lactate clearance is reduced to about half of normal after hemorrhage.

Although the proponents of the nutritive model admit that the hyperlactatemia of hemorrhagic shock probably has an anoxic basis, there are investigators that argue that, even in hemorrhage, hypoxia is not the sole cause of the increased lactate [145, 146]. They suggest that, as discussed in Septic Shock, glycolysis induced by increased catecholamines is the primary cause of the increased lactate. In support of this, alpha and beta blockers reduce the blood lactate increase by about 50% in a rat hemorrhage model [146]. Again, as we argued in Septic Shock, this could also be explained by a decrease in hypoxic tissue because of the block of the general thermogenic catecholamine action.

## 10. Discussion and Summary

The existence of lactate in human blood and its origin from ischemic muscle were, respectively, described about 240 and 130 years ago [147]. However, lactate was not commonly measured in clinical medicine until about 17 years ago when it was recognized that the blood level of lactate was a useful marker of the severity of disease, particularly with regard to various forms of shock. The recent appreciation of the high mortality of septic shock and the need for early aggressive intervention has led to the routine measurement of lactate in septic patients, with even a modest lactate elevation (>2 mM) serving as an indicator of the potential existence of shock. The goal of this review was to elucidate the quantitative pathophysiology underlying the strong correlation between blood lactate concentrations and mortality in various forms of shock in the hope of obtaining new insights into the etiology and treatment of shock. Unfortunately, as the above review makes clear, our detailed understanding of the factors associated with the increased blood lactate are so limited and uncertain that no definitive explanations are possible. In this section, we will try to summarize the issues and provide some tentative answers to the important questions.

Clearly, the most important issue is to distinguish between the two contrasting views of the generic cause of an increased blood lactate: the “nutritive” model versus the “hypoxic” model. These two models have markedly different pathophysiological implications. If the nutritive model is correct, the increased lactate is a beneficial response to the underlying pathology and “… a mechanism to mitigate the effects of injury and illness” [8]. An implication of this model is that Ringer's lactate is an ideal solute that should be used for fluid resuscitation [8, 148]. In contrast, if the hypoxic model is correct, blood lactate provides a valuable measure of the local tissue hypoxia and raises questions of why this hypoxia persists despite the increased cardiac output in septic shock, what tissues are primarily affected, and what therapeutic interventions (if any) should occur in response to persistent lactate elevation?

**Figure 8:**
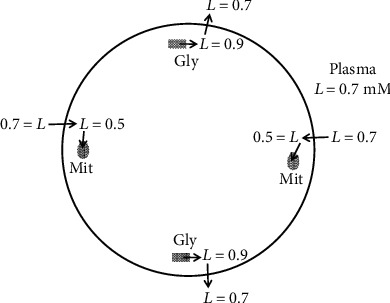
Schematic diagram of a radial cross section of a resting skeletal muscle cell illustrating net lactate production and release to the blood from glycogen (Gly, crosshatched squares) and simultaneous net lactate uptake from the blood and oxidation by the mitochondria (Mit, crosshatched circles). The lactate concentration is indicated by the symbol *L*. The simultaneous lactate uptake and release require that there are significant radial variations in the lactate cellular concentration adjacent to the plasma membrane.

A question that is directly related to this issue of hypoxic vs. nutritive models is how to interpret the observation from tracer lactate studies that there appear to be roughly equal rates of influx and efflux of lactate from some tissues (e.g., resting skeletal muscle, Table 3). It has been proposed that this bidirectional flux is evidence for the nutritive model because it indicates that two processes are occurring simultaneously in the same cell: (a) glycolysis programmed to produce pyruvate in excess of the ability of the local mitochondria to oxidize the pyruvate so that lactate is available for export from the cell to the blood where it is shuttled to other tissues and (b) lactate uptake from the blood that is converted to pyruvate, which “finds” mitochondria that are available to oxidize the pyruvate. It is essential for the validity of this simultaneous flux concept that these two processes occur in discrete, spatially separated areas such that intracellular lactate concentration at the glycolytic site exceeds that of serum allowing for the passive diffusion of lactate from the cell while lactate at the active mitochondrial site is less than plasma, allowing for the cellular influx of lactate. The distance between these two sites must be sufficient to prevent equilibration by diffusion of lactate, a requirement seemingly not compatible with the actual skeletal muscle histology.  Figure 8 schematically illustrates the distribution of glycogen (squares) and mitochondria (circles) in a cross section of a skeletal muscle cell at rest (plasma lactate = 0.7 mM). As discussed, the nutritive hypothesis requires significant radial variations in cellular lactate concentration adjacent to the plasma membrane to permit simultaneous passive influx and efflux of lactate. In actuality, the skeletal muscle has a very high, relatively uniform, density of mitochondria near the plasma membrane, representing more than 10% of muscle volume [149]. Assuming a mitochondrial diameter of 1 *μ* [150], the average separation between individual mitochondria is less than 2 *μ*. In addition, glycogen is densely packed in close contact with the mitochondria and uniformly distributed between the mitochondria [151]. These very short distances seemingly preclude the necessary lactate concentration differences required to simultaneously drive the passive lactate influx and efflux from the cell. During intense exercise, when the net efflux rate increases, the MCT transporters become rate limiting and the average cellular lactate concentration becomes appreciably greater than the plasma (see Exercise) and it is clearly impossible for some localized cellular region adjacent to the plasma membrane to have a concentration less than the plasma, as required for net uptake. These arguments effectively rule out this nutritive explanation of the simultaneous fluxes.

There are three other possible mechanisms that could contribute to the observed simultaneous unidirectional lactate uptake and release from, e.g., skeletal muscle. (1) An obvious explanation is that organs are histologically heterogeneous, with some cells consuming lactate and others releasing it. For example, in the skeletal muscle, type IIB muscle (white) fibers are primarily glycolytic and might be expected to release lactate while the oxidative type I fibers would oxidize lactate. However, this was not observed in direct measurements of net and tracer lactate fluxes in resting rabbit muscle [152], with both muscle types showing net lactate release. Thus, at least in resting conditions, this explanation of the simultaneous fluxes is not supported. (2) As discussed in The Krogh Model and Heterogeneity of Tissue O_2_ Partial Pressure, there is a well-established heterogeneity in the circulatory O_2_ supply to the cell, with oxidative metabolism occurring near the arterial end and hypoxic metabolism at the venous end. Thus, there could be a large longitudinal distance separating these two areas that would preclude lactate equilibration and would permit simultaneous lactate uptake and release at these two ends of the cell. (3) Finally, the simultaneous uptake and release of lactate may not represent distinct metabolic pathways, but rather, an artifactual result of rapid lactate-pyruvate exchange (see Lactate-Pyruvate Exchange).

The best quantitated aspect of lactate physiology is the whole-body steady-state uptake of labeled lactate (Table 1). This tracer flux suggests that lactate enters and is removed from the blood at a rate of 1.05 mmole/min/70 kg at rest. The organs responsible for this uptake are as follows: First, there clearly is net lactate uptake by the heart, kidney, and liver of about 0.48 mmole/min/70 kg (Table 3), accounting for about 46% of the tracer uptake. Although there could be some additional uptake either from cell heterogeneity or from the Krogh mechanism, these are probably of relatively minor importance, possibly accounting for another 10% or a total uptake of about 0.56 mmole/min/70 kg. The remaining 44% probably represents lactate-pyruvate “nonproductive” exchange. Note that, as predicted, this 44% is roughly the difference between the tracer and nonlabeled lactate uptake (Table 1).

Thus, at rest, the evidence suggests that there is net lactate uptake of about 0.56 mmole/min/70 kg balancing the net lactate release by the rest of the body organs (Table 3). There is no evidence to support the idea of an adjustable “lactate shuttle” at rest to meet varying energy requirements. In marked contrast, during exercise, there can be a large net shuttling of lactate between organs. This is clearly shown in, e.g., Figure 6(c), where the intensely exercising arm is releasing a huge net quantity of lactate (5.6 mmole/min/70 kg), all of which is being taken up by the recovering leg muscle. This observation does not, however, support the main thesis of the nutritive model that the lactate is being purposely released from the arm in order to provide a leg energy source. Rather, the simplest interpretation is that the intensely exercising arm muscle is working at an anaerobic exercise level that releases lactate. This arm release increases the blood lactate 10-fold to 7 mM, which diffuses into the recovering leg muscle, raising the muscle cell lactate concentration, which is driven by mass action in the direction of pyruvate oxidation or reduction to replenish glycogen expended during exercise.

Another argument used as support for the nutritive model is a negative one: that local tissue regions with the pO_2_ less than 1 mmHg necessary to impair pyruvate metabolism are not detectable. We have discussed in detail why this argument is also not conclusive (see The Krogh Model and Heterogeneity of Tissue O_2_ Partial Pressure). Because of the physiology of the tissue O_2_ supply (the Krogh model), the tissue pO_2_ is fundamentally heterogeneous and the quoted tissue pO_2_ measurements do not have the required resolution (10 microns) to detect the hypoxic regions. As discussed, the literature largely neglects studies carried out with the one technique, the O_2_ microelectrode, that has sufficient tissue resolution to identify localized areas of hypoxia. Studies with this microelectrode have shown that areas of tissue have a pO^2^ < 1 mmHg., i.e., hypoxic tissue consistent with the hypoxic model.

Findings specific to shock patients also seem to argue against the nutritive model. The proponents of the nutritive model have reasoned that septic shock, with its characteristic increased cardiac output, is a “hypermetabolic” state, similar to exercise, and the increase in blood lactate is produced by increased glycogenolysis in one tissue with the purpose of providing a circulating energy source for utilization in another tissue. However, as is illustrated in Table 1 and Figures 3 and 4, shock (septic, cardiogenic, and hemorrhage) differs markedly from exercise. Although there is an increase in whole-body lactate turnover in shock, this increase is about half of what would be predicted for normal subjects with the same increase in blood lactate and about one-fourth of that observed in exercise. That is, there is a decreased clearance (presumably from liver and kidney) in shock in contrast to the increased clearance in exercise. In addition, from the limited measurements that are available about individual organ blood flow and lactate flux in septic shock (Table 4), there is no significant increase in either muscle blood flow or lactate flux. Thus, there is no experimental support for the existence of the hypermetabolic state predicted by the nutritive model.

As discussed in Septic Shock, one would predict based on the hypoxic model that there should be an inverse correlation between lactate concentration and systemic vascular resistance (SVR) because, in response to hypoxia, the tissue will decrease its vascular resistance, increasing its blood flow. In contrast, for the nutritive model, one would not expect the SVA and lactate concentration to be correlated. Thijs et al. [109] described the time course of blood lactate and SVR in survivors versus nonsurvivors of septic shock. They found a strong inverse correlation between SVR and lactate, with the nonsurvivors having a lactate about 3 times greater and SVR about one-half, respectively, of the survivors. Bakker et al. [153] reported the dependence of the SVR and lactate on the “organ failure score” in septic shock patients. In going from the least to most severe score, the lactate doubled and the SVR fell in half. Thus, both these studies show the strong inverse correlation between SVR and blood lactate predicted by the hypoxic model.

Although there are quantitative data concerning net lactate balance in different organs in healthy humans (Table 3), surprisingly little is known about this balance in shock (Table 4). It is not possible to definitively state whether the increased blood lactate in septic shock is the result of increased production or decreased clearance because the only two available studies give conflicting results. Based on all the available human and animal results, it seems likely that both factors are contributing, with a combination of increased production and decreased clearance. If the hypoxic model is valid, the organ lactate production should provide a quantitative measure of the organ shock pathology, i.e., hypoxia. This is why it is so disappointing that, despite the enormous research effort, one cannot definitively identify the source of the increased lactate production in septic shock. The gut is the most likely source, as judged by its increased blood flow, with, e.g., a doubling of splanchnic blood flow, with little change in leg flow, in response to endotoxin in human volunteers [121]. That the hypoxic gut has the potential to produce the increased blood lactate in shock is clearly shown by the results in the pig, where a 60% isolated decrease in gut blood flow (with all other flows remaining normal) produced a 3-fold increase in gut lactate release and a 3.5-fold increase in blood lactate, similar to the values seen in shock [115].

Unfortunately, because of the difficulty of sampling the portal vein, there are no available measurements of gut lactate flux in human shock. There are a few measurements of splanchnic (gut plus hepatic) flux in human septic shock (Table 4) which indicate a net splanchnic uptake. As shown by the following quantitative model, if this net splanchnic uptake were less than normal because of the increased gut release, it could produce the increased blood lactate seen in septic shock. Assuming that during shock, the gut lactate release (*M*_gut_) increases while the rest of the body production (*M*_sys_) is constant and the hepatic (Cl_hep_) and renal (Cl_ren_) clearance decrease, the arterial blood lactate (*C*_a_) can be quantitatively described by

(3) $$\begin{array}{c} C_{\text{a}}=\frac{M_{\text{gut}}\left(F_{\text{ha}}+F_{\text{pv}}-{Cl}_{\text{hep}}\right)+M_{\text{sy}s}\left({\text{F}}_{\text{ha}}+F_{\text{pv}}\right)}{\left({Cl}_{\text{hep}}+{Cl}_{\text{ren}}\right)\left(F_{\text{ha}}+F_{\text{pv}}\right)}, \end{array}$$

where *F*_ha_ and *F*_pv_ are the hepatic artery and portal vein blood flow (assumed 0.45 and 1.1 L/min, respectively) [154]. Based on the data in Table 3, the normal values for *M*_sys_ and *M*_gut_ are 0.413 and 0.085 mmole/min, respectively, and Cl_ren_ and Cl_hep_ are 0.236 and 0.434 L/min, respectively. (Note: in this calculation, we are using only net uptake and are assuming that the difference between net and tracer is primarily exchange). Increasing *M*_gut_ 5-fold and halving the clearances increases *C*_a_ from 0.7 to 3.3 mM, similar to the changes observed in septic shock. Of special importance is that, despite the decrease in hepatic clearance and large increase in *M*_gut_, there is still a net splanchnic model uptake as is observed in human septic shock.

There is some indirect evidence that the gut is hypoxic in shock. Gastric tonometry measurements show that there is a significantly increased pCO_2_ and corresponding decreased mucosal pH_i_ in septic shock patients [155]. Although these changes are usually interpreted as resulting from hypoperfusion, it has also been postulated that the increased pCO_2_ results from buffering of the increased lactate and is a measure of hypoxia [156]. In a pediatric septic shock study, the decrease in the initial pH_i_ was better correlated with mortality than the blood lactate [157] and in a large series of adult septic shock patients, a persistent low pH_i_ despite treatment was strongly associated with a high mortality [158]. Gastric tonometry has also been used as guide for resuscitation in shock patients, but with only limited success [158–160].

This raises the critical question of why is the gut hypoxic despite the increased cardiac output and splanchnic blood flow in septic shock? It is now clearly established that there is a measurable dysfunction in the sublingual microcirculation that is correlated with the severity of the septic shock [120]. This results in an increased heterogeneity in the open capillaries leading to increased O_2_ diffusion distances and, presumably, an increase in local hypoxia. Whether one can extrapolate from sublingual observations to the gut is not known. The cause of this disruption is poorly understood, but is assumed to be related to the variety of inflammatory immune responses present in septic shock [161–164]. Haussner et al. [165] have recently summarized why the gut is particularly sensitive to sepsis and the myriad pathological events that can occur. In addition, sepsis is clearly a prothrombotic state that, at its extreme, can cause disseminated intravascular coagulation (DIC). Even in the absence of overt DIC, microvascular thrombi are thought to play an important role in organ dysfunction in sepsis and acute respiratory distress syndrome (ARDS).

Ultimately, given the limited quality of the available data, it is not currently possible to definitively determine all the potential contributors to hyperlactatemia in shock. Particularly with the severe metabolic derangements seen in septic shock, it seems likely that there are multiple causes that may exist simultaneously. However, the strong positive correlation between lactate levels and mortality and the lower mortality seen in patients with a decrease in lactate levels after initial resuscitation [105, 106] are more consistent with the prevailing theory that lactate levels are an important marker of tissue hypoperfusion.

In summary, we believe that the available experimental results support the classical “hypoxic” model in which the increase in blood lactate in shock provides a measure of the local tissue hypoxia. In cardiogenic or hemorrhagic shock, the hypoxia is caused by a decrease in tissue O_2_ delivery. In septic shock, with its increase in cardiac output, the hypoxia results from a distributive shock with critical redistribution of blood flow leaving some tissue beds (primarily gut) underperfused in the setting of microcirculatory disruption. The increase in the steady-state blood lactate is the result of a combination of an increase in lactate release and decrease in hepatic clearance. However, as we have tried to emphasize in this review, all of these statements are disputable because of the limitations in the quantitative experimental data.

## Figures and Tables

**Table 1:** Human whole-body circulatory lactate and glucose turnover, oxidation, and clearance. Two fundamentally different pharmacokinetic methods are listed: (1) unlabeled (“cold”) either as a “bolus” or as a “constant infusion” which measures the net lactate uptake and (2) tracer (either ^13^C or ^14^C) either as a bolus dose or as a constant infusion which measures lactate carbon exchange.

Ref	Method	Condition	Blood lactate (mM)	Lactate turnover (*μ*m/kg/min)	Glucose turnover	Lactate to CO_2_	Lactate to glucose	Clearance (L/min/70 kg)
[59]	^13^C bolus	Normal rest	0.84	23		49%		1.89
		Exercise light	1.5	45		81%		2.1
		Exercise mild	4.5	58		78%		0.9
[61]	^14^C constant	Normal rest	ND	18		85%	8%	
[58]	^14^C constant	Normal rest	0.65	15	11.4		21%	1.62
		Obese rest	0.6	12	7.65		22%	1.4
[63]	^14^C constant	Normal rest	0.72	14.4				1.4
		Exercise light	≈1.0	32.8				2.3
		Exercise mild	≈1.5	59.1				2.8
		Exercise heavy	≈4.0	120				2.1
[166]	^13^C constant	Normal rest	1.37	28	12.6			1.43
		Exercise mild	2.94	95	44.8			2.26
		Exercise heavy	5.22	136				1.83
	+ Infuse lactate	Normal rest	4.35	65	11			1.04
		Exercise mild	4.88	129	34.4			1.85
[57]	^14^C constant	Normal rest	0.6	10.3	9.8	61%	15%	1.2
		Diabetic	1.1	14.9	12.9	51%	17%	0.95
[60]	^13^C constant	Normal rest	0.9	11.2	7.2	65%	10%	0.84
		Septic shock	3.2	26.2	14.8	54%	15%	0.76
		Cardiac shock	2.8	26.6	15	43%	9%	0.68
[62]	^14^C constant	Normal rest	ND	11.2				
[64]	^13^C constant	Normal rest	0.9	16		45%		1.24
	+ Lactate	Rest	4.0	53		32%		0.93
	+ Lactate	Exercise	6.96	221		100%		2.23
[93]	^13^C constant	Rest	0.7	18.5		40%		1.85
		Exercise mild	2.5	214		90%		5.9
		Exercise heavy	7	357		70%		3.6
[67]	Cold bolus	Normal rest	0.7	13.2				1.32
[167]	Cold bolus	Normal rest	0.7	10.2				1.02
[168]	Cold bolus	Normal rest	0.53	7.4				0.98
[139]	Cold bolus	Normal rest	0.95	9.8	10.8	55%	18.5%	0.72
		Cardiac shock	6.7	33.6	29.5	61%	7.2%	0.54
[110]	Cold bolus	Normal rest	1.2	19.6				1.14
		Septic shock	2.6	19.9				0.53
[122]	Cold constant infusion	Normal rest	0.8	8.7				0.76
		Endotoxin shock	1.3	13.9				0.74

**Table 2:** Representative whole-body lactate and glucose turnover, oxidation, and interconversion. The values are the basal (resting, postprandial) values in normal human subjects.

	Lactate	Glucose
Blood Conc (mM)	0.7	4.9
Turnover (mmole/min/70 kg)	1.05	0.7
Clearance (L/min/70 kg)	1.5	0.142
Fraction oxidized to CO_2_	0.70	0.70
Fraction lactate converted to glucose	0.15	
Volume of distribution (L/kg body wt.)	0.45	0.25
Turnover time (min)	21	122

**Table 3:** Contributions of individual organs to the total basal resting postprandial human lactate uptake (*Q*) and release (*R*) in units of mmole/min. The gut value is extrapolated from the dog, all the rest are for human. If there are no tracer measurements, then only the net value is used for either *Q* or *R*, which are then a minimum estimate (indicated by the ≥ symbol), and it is assumed that there is no flux in the opposite direction (indicated by the ?? symbol). The net organ flux is listed in the last column (+ indicates net uptake, - indicates net release).

Organ	Reference	Uptake	Release	Net
Heart	[169]	0.067	0.025	+0.042
Brain	[64]	0.1	0.15	-0.05
Skeletal muscle	[64, 93, 170]	0.3	0.4	-0.1
Kidney	[29]	≥0.16	0^??^	+0.16
Liver	(See text)	≥0.28	0^??^	+0.28
Gut	(See text)	0^??^	≥0.085	-0.085
Adipose	[171–173]	0^??^	≈0.2	-0.2
Erythrocytes	(See text)	0	0.105	-0.105
Total		≥0.91	≥1.08	-0.056

**Table 4:** Blood lactate, cardiac index (CI), splanchnic and muscle blood flow, and splanchnic and muscle lactate net flux in human and animal shock models. The CI is expressed as the fraction of the normal resting CI (assumed equal to 3.3 L/min for humans). The organ flow is expressed as the fraction of the cardiac output. The fluxes are in units of *μ*mol/min/kg body wt. A positive flux corresponds to net uptake and negative to net release.

Reference	Species	Type	Lactate	CI	Splanchnic		Muscle	
			mM		Flow	Lactate flux	Flow	Lactate flux
[174]	Human	Septic	1.6	1.7	0.47	20		
[113]	Human	Septic	1.3	1.7	0.35		0.2	
[175]	Human	Septic	1.5	1.7	0.39			
[176]	Human	Septic	3.2	1.4	0.29			
[177]	Human	Septic	1.4	1.6	0.31	8.5		
[112]	Human	Septic	NA	1.37	0.24			
[118]	Human	Septic	3.8	1.21	0.3	13		
[178]	Dog	Endotoxin	4.2	0.78	0.21	-16	0.02	≈0
[179]	Dog	Endotoxin	NA	1.14			0.06	
[128]	Dog	Endotoxin	0.75					
[129]	Sheep	Septic	2.0	1.75	0.11 (1)			
[130]	Sheep	Endotoxin	10.2	1.4	0.32			
[132]	Pig	Septic	NA	0.85	0.4	80	0.09	≈0
[131]	Pig	Septic	1.25	1.0	0.24	12		
[131]	Pig	Tamponade	3.5	0.4	0.28	-5.1		
[180]	Pig	Endotoxin	NA	1.0	0.24		0.1	
